# Surgical management of growing teratoma syndrome requiring cardiopulmonary bypass and total superior vena cava resection and reconstruction

**DOI:** 10.1186/s13019-025-03813-z

**Published:** 2026-01-05

**Authors:** Elaine Liang, Barkha Trivedi, Dominic Amara, Jeffrey B. Velotta

**Affiliations:** 1 Kaiser Permanente Bernard J. Tyson School of Medicine, Pasadena, CA USA; 2 University of California Los Angeles, Los Angeles, CA USA; 3 Department of Surgery, University of California Los Angeles, Los Angeles, CA USA; 4 Department of Surgery, University of California San Francisco, School of Medicine, San Francisco, CA USA; 5 Department of Thoracic Surgery, Kaiser Permanente Oakland Medical Center, Oakland, CA USA

**Keywords:** Mature teratoma, Growing teratoma syndrome, Cardiopulmonary bypass, Vascular graft reconstruction, Superior vena cava, Non-seminomatous germ cell tumors

## Abstract

**Background:**

After chemotherapy, a small subset of mediastinal non-seminomatous germ cell tumors evolves into growing benign teratoma masses despite normalized tumor markers. This condition is termed the growing teratoma syndrome. We present a case of a massive mediastinal mature teratoma requiring cardiopulmonary bypass and complete vascular graft reconstruction of the superior vena cava and innominate vein confluence.

**Case presentation:**

A 31-year-old man presented to the emergency department with acute right-sided chest pain, dyspnea, and weight loss. Imaging revealed a 16.7 cm anterior mediastinal mass compressing the superior vena cava, with elevated alpha-fetoprotein and beta-human chorionic gonadotropin levels. Biopsy results revealed mature teratoma. After three cycles of platinum-based chemotherapy, tumor markers normalized, but the mass enlarged to 22 cm. He then underwent clamshell thoracosternotomy with cardiopulmonary bypass, mediastinal mass complete en bloc resection, lung sparing, and venous graft reconstruction of the superior vena cava. Final pathology confirmed mature teratoma with negative margins.

**Conclusions:**

This case highlights the importance of recognizing growing teratoma syndrome in patients with an enlarging mediastinal mass despite normalized tumor markers following chemotherapy for presumed non-seminomatous germ cell tumor. Complete surgical resection remains the only curative treatment, though it is highly technically challenging. Early surgical referral and multidisciplinary planning are essential for optimal outcomes in such high-risk cases.

## Background

Non-seminomatous germ cell tumors (NSGCTs) are a heterogeneous group of malignancies that contain a mix of histologies, including teratoma, embryonal carcinoma, yolk sac tumor, and choriocarcinoma. They are diagnosed by elevated serum tumor markers, alpha-fetoprotein (AFP) and beta-human chorionic gonadotropin (β-hCG), which are unique to NSGCTs and essential for prognosis and treatment [1]. Patients with elevated markers receive 3–4 cycles of platinum-based chemotherapy, such as BEP (bleomycin, etoposide, and cisplatin) or VIP (etoposide, ifosfamide, and cisplatin) [2].

In contrast, teratomas are germ cell tumors composed of tissue from at least two embryonic germ layers. Mature teratomas contain well-differentiated tissue, are usually benign, but are often resistant to chemotherapy [2]. In NSGCTs, residual mature teratoma may continue to grow after chemotherapy even when tumor markers fall or normalize. This phenomenon was first described by Logothetis et al. in 1982 as “growing teratoma syndrome” (GTS) [3]. The etiology of GTS is unclear, but proposed mechanisms include chemotherapy eradicating malignant immature cells while sparing mature teratomatous elements, or inducing differentiation of totipotent malignant germ cells into benign teratoma [4]. We report a case of mediastinal NSGCT transforming into GTS in a healthy 31-year-old man without metastases.

## Case presentation

A 31-year-old male with no significant medical history presented to the emergency department with acute right-sided chest pain and a two-week history of cough. He described initial cold-like symptoms, worsening dyspnea, and a 10-pound weight loss that had not improved after recent travel to Egypt. He reported smoking vapes and drinking alcohol but denied other drug use. Tumor markers revealed highly elevated alpha-fetoprotein (10,655 ng/mL) and β-human chorionic gonadotropin (612 mIU/mL), but normal lactate dehydrogenase (137 U/L). Transthoracic echocardiogram revealed right atrial compression from the mediastinal mass with otherwise normal ejection fraction (EF) and no evidence of pulmonary arterial hypertension.

**Fig. 1:**
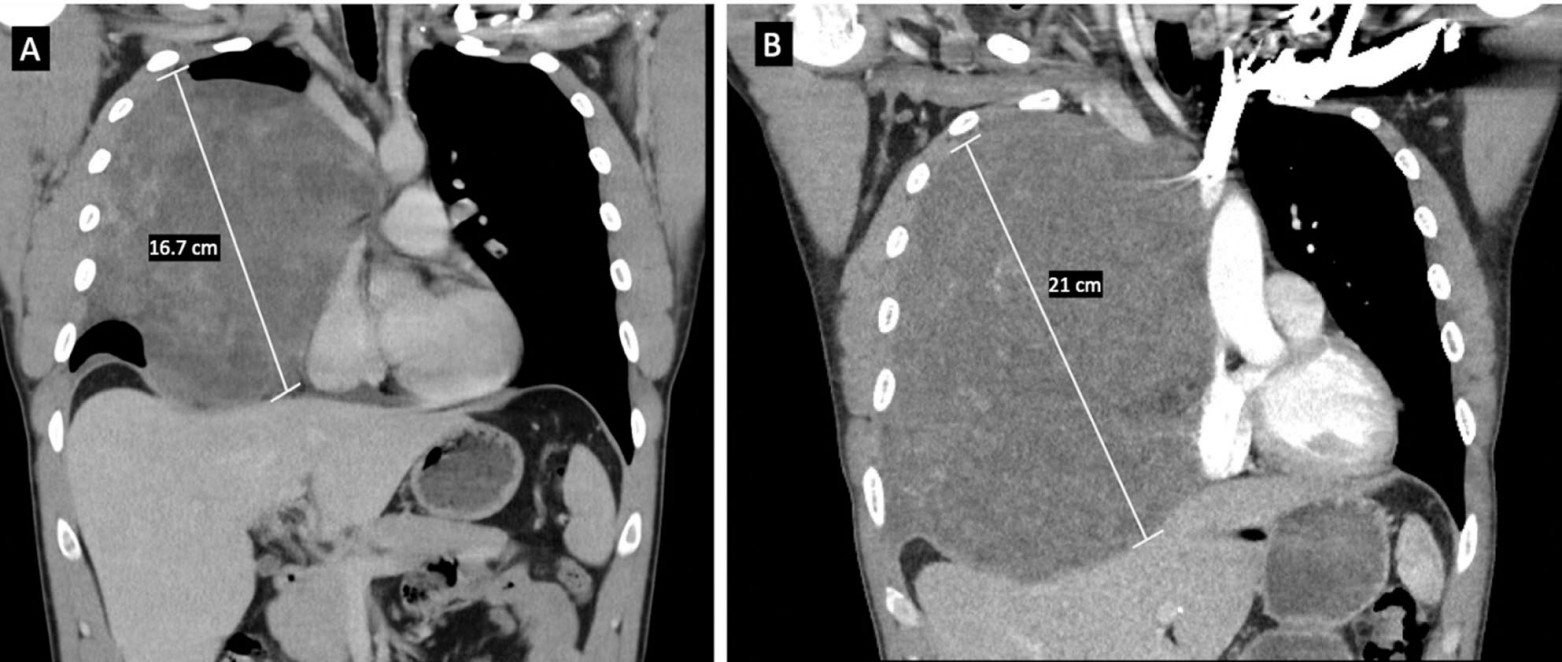
Chest computed tomography angiography (CTA) showing **(A)** initial 16.7 cm mass with smooth margins and compressed SVC. **(B)** Repeat chest CTA showing 21 cm mass after completing chemotherapy 2 months later

Chest computed tomography angiography (CTA) revealed a 16.7 cm right anterior mediastinal mass with smooth margins, causing leftward displacement of the heart and mediastinum and severe compression of the superior vena cava (SVC) **(**Fig. 1A**)**. Abdominal magnetic resonance imaging (MRI) demonstrated multiple hepatic hemangiomas and partial visualization of the mediastinal mass. CT-guided core biopsy revealed a mature teratoma but given the lesion’s large size and elevated AFP and β-hCG levels, additional germ cell tumor components consistent with NSGCT could not be excluded.

Although pathology raised concern for a mixed germ cell tumor, particularly an NSGCT of testicular origin, scrotal ultrasound showed no testicular mass. A brain MRI was also obtained to rule out metastases and came back negative. Given the tumor’s large size, encasement of the SVC, and possible NSGCT identity, two multidisciplinary tumor boards recommended upfront chemotherapy rather than surgery. Surgical resection was considered only if the patient became significantly symptomatic and the procedure can be safely performed, but initial chemotherapy was preferred to reduce tumor size. The patient tolerated three cycles of VIP chemotherapy (etoposide, ifosfamide, and cisplatin) well, with symptomatic improvement and downtrending tumor markers.

However, repeat chest CTA following completion of chemotherapy two months later showed an increase in the right mediastinal mass size from 16.7 cm to 21 cm **(**Fig. 1B**)**. The mass abutted the mediastinum and pericardium without evidence of invasion into the chest wall or diaphragm. The patient’s case was again reviewed by the multidisciplinary tumor board, who recommended against further chemotherapy and radiation. Surgery remained as the only viable treatment option despite a high morbidity and mortality risk due to superior vena cava involvement. The patient was fit and still minimally symptomatic, with disease progression confined to the chest and no distant metastases.

**Fig. 2:**
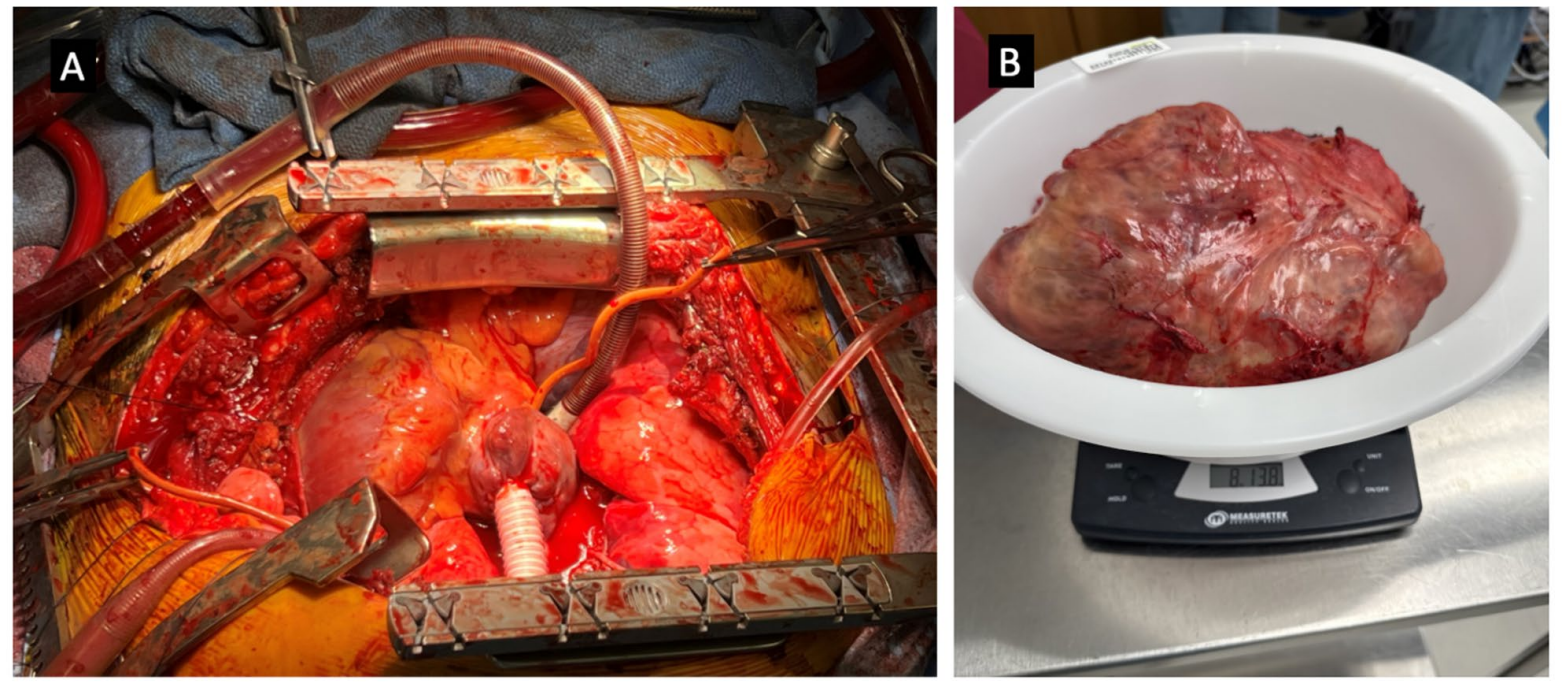
Intraoperative gross appearance of the mediastinal mass. **(A)** Cardiopulmonary bypass done to safely resect the mass from the right hemithorax. **(B)** The final resected right mediastinal teratoma, measuring 23.0 × 21.0 × 13.0 cm and weighing approximately 8 lbs, 13 oz. (3997 g)

**Fig. 3:**
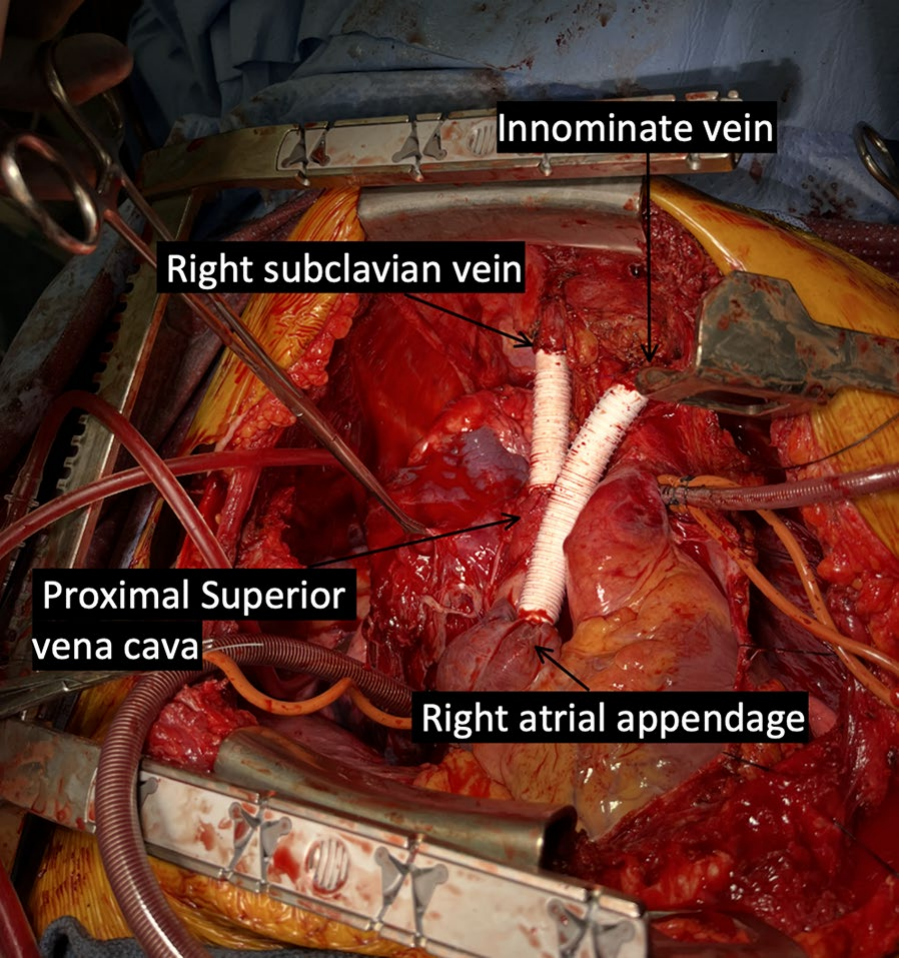
Final reconstruction of the first Gore-Tex graft anastomosing the proximal SVC to the right subclavian vein and second Gore-Tex graft anastomosing the right atrial appendage to the innominate vein

The preoperative workup included a V/Q scan (left lung: 96%, right: 4%), pulmonary function tests (FEV1: 53%, DLCO: 75%), and repeat CT showing tumor growth from 21 cm to 22.4 cm over 2.5 weeks. AFP decreased from 236 to 77 ng/mL. Surgery included bilateral thoracosternotomy (clamshell incision), cardiopulmonary bypass **(**Fig. 2A**)**, complete en bloc resection of the mass along with SVC resection and reconstruction with Gore-Tex PTFE graft. Specifically, the tumor occupied the right hemithorax and invaded the confluence of the right subclavian and innominate veins (origin of the SVC), requiring reconstruction of these vessels using 14 French ringed Gore-Tex PTFE grafts under cardiopulmonary bypass. Cardiopulmonary bypass was initiated earlier than the SVC reconstruction due to the tumor’s extensive size and involvement with the SVC. Complete (R0) resection could not be achieved without compromising venous return and causing hemodynamic instability. Therefore, bypass was performed to facilitate safe tumor excision rather than for the SVC reconstruction itself. Once on bypass, the subsequent SVC and innominate vein reconstruction were performed efficiently and safely, allowing careful dissection of the mass from the right lung. Venous reconstruction was performed with end-to-end anastomoses of Gore-Tex grafts to the proximal SVC, right subclavian vein, innominate vein, and right atrial appendage **(**Fig. 3**)**. SVC reconstruction was performed on low flow moderate hypothermic cardiopulmonary bypass with minimal clamping time.

Bilateral intercostal cryoablation was performed prior to chest closure. 4 chest tubes (3 right and 1 left pleural) were placed. Cardiopulmonary bypass time was 159 min. The patient received transfusions of 6 units of red blood cells, 2 units of platelets, and 10 units of cryoprecipitate, with additional hemostatic support using 1000 units of intravenous Kcentra. The chest was closed using a Stryker plating system to reappose the sternal body. The patient was transferred to the cardiovascular intensive care unit (CVICU) in stable condition.

Overall, the patient had an uneventful postoperative recovery and was discharged home on postoperative day 8. At discharge, he had normal cardiac function and was continued on metoprolol 12.5 mg twice daily for rate control. He was also started on dual antiplatelet therapy with aspirin 81 mg daily and clopidogrel for one year, with final duration beyond that period at the discretion of cardiology, and rivaroxaban for three months to manage a right internal jugular vein thrombosis. Wound care instructions and colchicine prophylaxis for post-pericardiotomy syndrome were provided. Final pathology confirmed a mature teratoma with R0 resection. The specimen measured 23.0 × 21.0 × 13.0 cm and weighed 8 lbs 13 oz (3997 g) **(**Fig. 2B**)**. His tumor markers have normalized, and he is currently on a surveillance imaging protocol every 6 months.

### Discussion and conclusions

The anterior mediastinum is the most common site of extragonadal germ cell tumors, with over half classified as mature teratomas. Among malignant tumors, 40% are seminomas and 60% are NSGCTs [5]. Mediastinal NSGCTs are found almost exclusively in young males and tend to grow rapidly, often compressing mediastinal structures like major blood vessels. They respond well to platinum-based chemotherapy, typically resulting in tumor shrinkage and normalized AFP and β-HCG serum levels. However, approximately 3–8% of testicular NSGCTs with normalized tumor biomarkers develop GTS [6].

GTS is defined by three criteria: (1) previously elevated tumor markers, namely AFP and β-HCG, have normalized after chemotherapy, (2) the tumor size increased after chemotherapy with the presence of compression symptoms, and (3) final pathology confirmed only mature teratoma without any other NSGCT components [7]. Although several cases of GTS have been described [3, 4, 6–9], its natural history remains unclear and physician awareness of this syndrome is limited [9]. GTS has been previously described to be located in the retroperitoneum (the most common site), iliac nodes, mediastinum, supraclavicular/cervical lymph nodes, inguinal lymph nodes, lung pleura, forearm, liver, mesentery, and pineal gland [4, 9]. Once identified, the recommended treatment is immediate cessation of chemotherapy followed by an early and complete surgical resection of the tumor [10].

In our case, despite limited awareness of GTS, the growing mediastinal mass with normalized tumor markers suggested a refractory tumor, prompting immediate cessation of VIP chemotherapy. The main viable option is surgery, and it remains the only curative treatment option [7]. Complete R0 mediastinal GTS resection is very technically complex with a 4% operative mortality secondary to pulmonary complications and high morbidity as it involves the lung, phrenic nerve, great vessels, and cardiac chambers. Postoperative care requires meticulous fluid management and respiratory support [11]. The recurrence rate of GTS following complete surgical resection is as low as 4%, but incomplete resection is as high as 83% [7]. Even with treatment, prognosis remains poor, with a 5-year survival rate of 30–48% [7].

Despite the risks and technical challenges, we proceeded with surgery due to the patient’s overall excellent physical status and disease confined to the mediastinum without evidence of distant metastases. Surgical approaches for mediastinal GTS have included clamshell/hemiclamshell thoracosternotomy, anteroposterior thoracotomy, median sternotomy, video-assisted thoracoscopic surgery (VATS), posterolateral incision, and simple neck collar incision. Depending on the size, location, and invasion of the tumor, surgery may also involve anatomical lung resection, pericardiectomy, phrenic nerve resection, and ligation of the innominate vein [6, 7, 11, 12]. Given the large size of the tumor occupying the right hemithorax with compression towards the left chest, we opted for the bilateral thoracosternotomy (clamshell incision). The patient was put on full cardiopulmonary bypass support to allow for complete resection of the SVC and part of the innominate vein with full vascular graft reconstruction. In addition, the entire right lung was spared with R0 resection margins. In addition to ours, there is only one other reported case report of a 20 cm mediastinal teratoma that similarly required a bilateral clamshell thoracotomy, SVC reconstruction and cardiopulmonary bypass [13].

When considering GTS treatments, we support an aggressive surgical approach to achieve margin-negative resection as it offers a potential cure for localized tumors. This case shows that even massive tumors can be safely removed with careful planning and multidisciplinary coordination, minimizing morbidity and optimizing patient outcomes. To our knowledge, this case represents one of the largest resected mediastinal GTS tumors documented, requiring cardiopulmonary bypass and a full vascular graft reconstruction of the SVC. We achieved complete (R0) resection of the teratoma with negative margins, and this is typically curative for lesions that are mature teratoma on pathology [11]. The patient had an uneventful recovery, is doing very well, and stays active by hiking and biking routinely. Overall, GTS should be suspected in patients with growing tumors despite normalized NGSCT markers, and early and aggressive surgical salvage resection should be the first-line treatment.

## Data Availability

All data generated or analyzed are included in this article.

## Abbreviations

NSGCTs: Non-seminomatous germ cell tumors
GTS: Growing teratoma syndrome
AFP: Alpha-fetoprotein
β-hCG: Beta-human chorionic gonadotropin
LDH: Lactate dehydrogenase
BEP: Bleomycin, etoposide, and cisplatin chemotherapy regimen
VIP: Etoposide, ifosfamide, and cisplatin chemotherapy regimen
CTA: Chest computed tomography angiography
SVC: Superior vena cava
CT: Computed tomography
MRI: Magnetic resonance imaging
VATS: Video-assisted thoracoscopic surgery
FEV1: Forced expiratory volume per second
DLCO: Diffusing capacity of the lung for carbon monoxide
V/Q: Ventilation/perfusion scan
PTFE: Polytetrafluoroethylene
EF: Ejection fraction
CVICU: Cardiovascular intensive care unit
